# Erectile Dysfunction: Causes, Diagnosis and Treatment: An Update

**DOI:** 10.3390/jcm11216429

**Published:** 2022-10-30

**Authors:** Fernando Mazzilli

**Affiliations:** 1 Andrology Unit, Department of Clinical and Molecular Medicine, “Sapienza” University of Rome, Sant’Andrea Hospital, Via di Grottarossa 1038, 00189 Rome, Italy; fernando.mazzilli@uniroma1.it or fernando.mazzilli50@gmail.com; 2 AIED Center for Reproductive Medicine, Via Toscana 30, 00187 Rome, Italy

## 1. Introduction

Erectile Dysfunction (ED) is defined as “recurrent and persistent inability, partial or complete, to achieve or maintain an erection firm enough for satisfactory sexual intercourse in the presence of proper erotic stimuli”; the concept of ED replaced the previous definition of “impotence” [1]. ED should be considered a social problem with the potential to affect men of all ages and ethnicities and have a significant impact on the quality of life (QoL) of both the patient and his partner [2]. Epidemiological data estimate a prevalence of ED between 14% and 48%. The width of this range is probably due to methodological differences between studies regarding age and socio-economic status [3]. Moreover, the prevalence could be underestimated due to male privacy and reservedness. The ability to have and maintain an erection as a voluptuary act is the prerogative of man and does not exist in many other animal species. Age plays an extremely important role in maintaining healthy sexual activity. In fact, the overall age-specific prevalence of moderate or complete ED ranges from 9% in men aged 40–44 years up to 56% in men over 65 years [2]. Considering lifestyle habits, cigarette smoking, alcohol abuse and recreational drug use negatively affect the quality of sexual function. Distress, which refers to the negative aspect of stress, also affects sexual activity. Even a condition of prolonged infertility can cause sexual difficulties, especially during the partner’s ovulatory period, where “on command” intercourse is indicated [4].

## 2. Causes of ED

The causes of ED can be distinguished as those occurring with male hypoactive sexual desire disorder and those with normoactive sexual desire. The first condition can sometime depend on a diminished attraction towards one’s partner, which can result from illnesses or simply from a prolonged duration of the couple’s relationship. Furthermore, it can be related to psychogenic conditions or organic diseases. Psychogenic conditions are frequently related to misunderstandings within the couple or the family unit, as well as problems related to work activities, that can frequently affect sexual desire. Even the occurrence of initial episodes of ED, as well as other sexual dysfunctions, can lead to performance anxiety and thus an evasive reaction to avoid failure. Among organic diseases, the most important endocrine cause is a condition of hypogonadism; in fact, testosterone is the main driver of sexual desire. Hyperprolactinemia also causes a decrease in sexual desire and, therefore, in sexual performance; finally, hypothyroidism can cause a decrease in desire, probably due to hyperprolactinemia resulting from hypothyroidism [5]. Among psychiatric causes, certainly depression, which is characterized by an inability to “feel pleasure” and a mood oriented towards sadness, represent a cause of ED [6]; other chronic systemic diseases can lead to a deterioration in the QoL and therefore a decreased desire for sex.

The other condition (ED with normoactive sexual desire) can be due to vasculogenic, cardiovascular and metabolic diseases, as well as neurological, inflammatory/infectious, and mechanical and iatrogenic causes. These pathogenic conditions lead to a deficit in the synthesis and release of vasodilating agents, including NO, which is essential for filling the corpora cavernosa. Therefore, ED and cardiovascular diseases (CVD) should be regarded as two different manifestations of the same systemic disorder. ED usually precedes CVD onset, and it might be considered an early marker of symptomatic CVD, as well as of DM [7,8,9]. It has also been shown that DM can cause sexual dysfunction in women [10]. Neurogenic causes, such as multiple sclerosis, Parkinson’s disease and spinal cord injuries are mainly characterized by failure to initiate sexual intercourse. Furthermore, patients undergoing radiotherapy and radical pelvic surgery (i.e., radical prostatectomy) have a high risk of lesions of the cavernous nerves with consequent neurogenic ED. Mechanical conditions are mainly due to cavernous fibrosis due to induration penis plastica (IPP) or following penile trauma. In patients affected by male accessory gland infection (MAGI) and presenting ED symptoms, a dilation of the periprostatic venous plexus was evidenced [11]. Recently, ED was also reported as a sequel to a previous infection from COVID 19.

Finally, even some categories of drugs, such as anti-androgens, H2-antagonists, ACE-inhibitors and psychotropic drugs, can cause ED via mechanisms not completely known.

## 3. Diagnosis

The presence and severity of ED should be assessed through a careful anamnestic investigation (onset of ED, time from onset, correlation with a specific partner, couple conflicts, erection/rigidity, ejaculation without erection, nocturnal spontaneous erections) and through specific questionnaires (i.e., International Index of Erectile Function- IIEF 15 (ED: total score < 26) or- IIEF 5 (ED: total score < 22). There are various degrees of impairment, according to IIEF score (mild, moderate and severe) [12]. In addition, specific investigations of first-level (hormonal and biochemical) and second-level (penile color Doppler, monitoring of nocturnal penile erections and neurogenic reflex examination) can be indicated. Obviously, the psychosexual approach is essential. Furthermore, in about 10% of the sexually healthy population, occasional or repetitive episodes of difficulty getting/maintaining erection can occur [13]. This can be due to performance anxiety and then sexual discomfort; in such situations, the definition of subclinical erectile dysfunction has been proposed [14].

## 4. Treatment

In previous centuries, ED treatment was based on alchemy and aphrodisiacs. At the beginning of the 20th century, Freud’s pan-psychic hypothesis was proposed. Therefore, until the 1970s, the only alternative to psychotherapy was the administration of androgens. Currently, lifestyle correction constitutes an essential therapeutic approach. The second approach concerns, where possible, the treatment of known etiopathogenic causes. Considering endocrine and metabolic pathologies, the treatment of hypogonadism consists of correction through testosterone replacement therapy. Furthermore, hyperprolactinemia and hypo- or hyperthyroidism should be corrected. The control of the lipid/metabolic profile is also essential to recovering acceptable sexual performance. A particularly complex situation concerns the interference of drugs in the erective mechanism or in sexual desire, especially in those cases in which it is not possible to suspend or replace the drug. Obviously, the psychosexual approach remains effective and indispensable in cases of relational and intrapsychic causes, in order to stimulate the recreational and hedonistic aspect of sexuality.

### 4.1. PDE5-i Treatment

Oral phosphodiesterase type 5 inhibitors (PDE5i) are the primary pharmacologic treatment for erectile dysfunction (ED) [15]. Sildenafil was first studied in clinical trials for coronary heart disease, but it was occasionally observed that the drug had favorable effects on penile erections. Therefore, by 1998, Sildenafil was FDA approved as the first oral treatment for ED [16]. In the body, there are 11 distinct PDE isoenzymes expressed in different concentrations in various tissues. The PDE5 enzyme is widespread, but is more prevalent in penile tissue. In addition to PDE5 inhibition, sildenafil also weakly inhibits PDE6, an enzyme present at high concentrations at the retinal rod and cone photoreceptors, potentially leading to mild impairment of color discrimination [17]. For this reason, the first PDE5i was defined as the blue pill.

Endothelial cells produce nitric oxide (NO), which activates the guanylate cyclase enzyme, inducing the conversion of guanosine triphosphate (GTP) to cyclic guanosine monophosphate (cGMP). Consequently, there is a decrease of intracellular calcium ions in the cavernosal smooth muscles, leading to smooth muscle relaxation, increased arterial blood flow and venous constriction, thus reducing the drainage of arterial blood and sustaining the erection [18].

Generally, at the end of sexual intercourse, the action of PDE5 takes over, which inactivates cGMP and transforms it into GMP, causing penile flaccidity. Depending on age, stress and other factors, the action of PDE5 can occur earlier and inappropriately. Therefore, the action of the PDE5i consists of inhibiting this last stage, thus allowing the persistence of a valid erection. Thus, these drugs do not induce a “mechanical” erection, but ameliorate the action of NO. To achieve tangible efficacy, both sexual desire and attraction for the sexual partner must be present, as well as the absence of stressful factors.

To date, four PDE5is are globally available (sildenafil, tadalafil, vardenafil and avanafil); on the other hand, mirodenafil, udenafil and lodenafil are only available in certain countries. The formulations are of various types (pills, films), each of them with specific dosages. The mechanism of action is similar for all PDE5i, but the pharmacokinetic properties are different for each of them. Therefore, consideration should be given to the time to maximum concentration in the blood (Tmax), the onset of action, the plasma half-life (T1/2), and the duration of action.

These properties can be summarized as follows: (a) Sildenafil: Tmax: 60’; onset of action: 30’–60’; T1/2 4 h; action duration: 12 h; (b) Tadalafil: Tmax: 120’; onset of action: 15’–45’; T1/2 17.5 h; action duration: 36 h; (c) Vardenafil: Tmax: 60’, onset of action: 15’–30’; T1/2 4–5 h; action duration: 12 h; ( d) Avanafil: Tmax: 30’–45’; onset of action: 15’; T1/2 3–5 h; action duration: 6 h. A meal rich in fat prolongs the duration of action of these drugs, apart from that of tadalafil [19].

PDE5i are generally used “on demand”. In 2008, tadalafil earned the first market approval for once-daily dosing, which allows for a constant plasma concentration and thus may appeal to individuals desiring increased spontaneity. The choice of a drug must therefore be adapted as much as possible to the patient’s needs.

The efficacy depends on the dosage used, as well as the individual response, but also the hemodynamic characteristics of the individual PDE5i. A number of trials have examined the efficacy and safety of PDE5i with regard to objective efficacy parameters and subjective patient preference. The results of a meta-analysis indicated that all of the oral PDE5 inhibitors were more effective than a placebo in all studied domains of ED. Currently, there is no clear evidence that one PDE5i is more effective than the others [20].

Furthermore, continuous PDE5 inhibition could offer a strategy to target cardiorenal complications of T2DM, with sex- and tissue-specific responses [21].

The most commonly reported complications of oral PDE5 inhibitors are headache, flushing, dyspepsia, dizziness and rhinitis. Oral PDE5 inhibitors have slight off-site binding affinity to other PDE enzymes. Vasodilation and cardiovascular safety are pertinent concerns that are reflected in the drugs’ contraindications, warnings and precautions. Therefore, any concurrent use of organic nitrates, including sublingual nitro-glycerine, nitrite and isosorbide mononitrate or dinitrate, is contraindicated.

### 4.2. Nutraceuticals

A recent meta-analysis [22] evaluated numerous randomized controlled trials on the use of nutraceuticals (ginseng, saffron, *Tribulus terrestris*, *Pinus pinaster* and *Lepidium meyenii*) or dietary supplements in patients with ED. L-arginine as well as L-citrulline has also been proposed as a nutritional supplement for ED. The mechanism of action remains unclear, but each of these appears to partially increase NO synthesis [23].

However, further larger and high-quality studies are required before firm conclusions can be drawn [24]. 

### 4.3. Intracavernous Drug Administration

For patients unresponsive to medical and lifestyle treatments, several second- and third-line therapies are available [19]. Intracavernous drug administration use dates back to the early 1980s; in fact, the availability of new diagnostic tools such as Doppler made it possible to discover the pro-erectile activity of papaverine and phentolamine administered within the corpora cavernosa, which led to a real revolution in the approach to patients with ED. These two substances were then replaced by Alprostadil (PGE1), which increases cAMP levels through adenylate cyclase stimulation, leading to smooth cell relaxation, vasodilation and penile erection. Currently, pharmacoerection with PGE1 is used in situations with endothelial damage characterized by a reduction in the availability of NO. Since the presence of several complications, such as priapism and fibrosis after PGE1 injections, intraurethral devices and cream alprostadil have been proposed as alternative routes of administration.

### 4.4. Physical Treatments

Physical treatments for ED management have been proposed over time. For example, the “vacuum device” is a cylindrical mechanical device that is placed around the penis and pumped; consequently, it creates a negative pressure vacuum to draw blood into the penis. The reported adverse events included penile pain, numbness and bruising. Furthermore, over the past decade, low-intensity extracorporeal shockwave therapy (Li-ESWT) has emerged as a treatment modality for ED [25].

### 4.5. Surgical Treatment

Surgical techniques of penile revascularisation were developed in order to anastomose the inferior epigastric artery to either the dorsal artery or deep dorsal vein, to improve penile vascular inflow while reducing venous outflow. However, these techniques did not meet with much success.

Actually, among the surgical approaches for ED, penile prosthesis is an attractive and effective option for patients unresponsive to medical treatments. Of course, this approach represents a permanent solution to the problem.

Penile implants include malleable and inflatable devices. The malleable implant consists of two semi-rigid rods that are placed in the corpora cavernosa. On the other hand, two-piece inflatable penile prostheses involve two cylinders with a scrotal pump, which allows fluid to be transferred to the cylinder chambers when an erection is desired [26].

## 5. Future Perspectives

The race to replace PDE5i or other actual therapeutic approaches can be identified as evident from the significant number of patents filed and the inventions cleared with clinical trials [27]. Finally, there is considerable interest in understanding the genetics of erectile dysfunction. It has been reported that Genome-Wide Association Studies can identify a risk locus for erectile dysfunction [28]. These findings could contribute to the development of future therapies for this disorder [29].
